# Megakaryocytes Are Regulators of the Tumor Microenvironment and Malignant Hematopoietic Progenitor Cells in Myelofibrosis

**DOI:** 10.3389/fonc.2022.906698

**Published:** 2022-05-11

**Authors:** Lilian Varricchio, Ronald Hoffman

**Affiliations:** Tisch Cancer Institute, Icahn School of Medicine at Mount Sinai, New York, NY, United States

**Keywords:** tumor microenvironment, hematopoietic stem cells, megakaryocytes, cytokines, myelofibrosis

## Abstract

Megakaryocytes (MKs) are multifunctional hematopoietic cells that produce platelets, serve as components of bone marrow (BM) niches that support the development of hematopoietic stem and progenitor cell (HSPC) and provide inflammatory signals. MKs can dynamically change their activities during homeostasis and following stress, thereby regulating hematopoietic stem cell (HSC) function. Myelofibrosis (MF) is a progressive chronic myeloproliferative neoplasm (MPN) characterized by hyperactivation of JAK/STAT signaling and MK hyperplasia, which is associated with an aberrant inflammatory signature. Since JAK1/2 inhibitor alone is incapable of depleting the malignant HSC clones or reversing BM fibrosis, the identification of mechanisms that cooperate with MF JAK/STAT signaling to promote disease progression might help in developing combination therapies to modify disease outcomes. Chronic inflammation and MK hyperplasia result in an abnormal release of TGFβ1, which plays a critical role in the pathobiology of MF by contributing to the development of BM fibrosis. Dysregulated TGFβ signaling can also alter the hematopoietic microenvironment supporting the predominance of MF-HSCs and enhance the quiescence of the reservoir of wild-type HSCs. Upregulation of TGFβ1 levels is a relatively late event in MF, while during the early pre-fibrotic stage of MF the alarmin S100A8/S100A9 heterocomplex promotes pro-inflammatory responses and sustains the progression of MF-HSCs. In this review, we will discuss the recent advances in our understanding of the roles of abnormal megakaryopoiesis, and the altered microenvironment in MF progression and the development of novel combined targeted therapies to disrupt the aberrant interplay between MKs, the BM microenvironment and malignant HSCs which would potentially limit the expansion of MF-HSC clones.

## Introduction

Myelofibrosis (MF) is a clonal blood cancer originating at the level of the hematopoietic stem cell (HSC) which is characterized by the acquisition of specific MPN driver mutations in janus kinase 2 (*JAK2)*, calreticulin (*CALR)*, and myeloproliferative leukemia virus oncogene (*MPL*) resulting in constitutive activation of JAK-STAT signaling (1). Although allogeneic stem cells transplantation can eradicate malignant MPN cells resulting in cure, most MF patients are not candidates due to co-morbidities and advanced age while other commonly used treatment options have limited effects on patient outcomes.

The evolution of MF is associated with development of profound bone marrow (BM) fibrosis (reticulin and/or collagen deposition), progressive splenomegaly, systemic symptoms, anemia and thrombocytopenia. Despite MF has being considered a chronic disease, the median expected survival is 5.9 years (2).

Abnormalities in megakaryocyte (MK) numbers and morphology are considered the common feature of the MPNs. MK morphology plays a critical role in distinguishing MF from other types of Philadelphia-negative MPNs such as polycythemia vera (PV) and essential throbocythemia (ET) (3, 4). MK is the largest (50-100 µm) hematopoietic cell within the BM. Under physiological conditions, MK precursor cells after multiple rounds of endomitosis become polyploidy (5) undergo further terminal maturation and then generate platelets necessary for normal homeostasis and coagulation. BM histopathologic changes in MF include MKs hyperplasia, tight clustering, small size and hypolobulated nuclei accompanied by fibrosis. Several studies have used ex vivo culture systems with purified MF CD34+ or mononuclear cells to generate increased number of MKs, which remained immature with reduced ploidy (3, 6). Moreover, MF-MKs display impaired pro-platelet formation when compared with MKs derived from PV, ET and healthy donors (3, 7).

Alterations in MK maturation lead to excessive release of pro-inflammatory cytokines and growth factors, which alter the hematopoietic microenvironment in a manner that supports MF stem/progenitor cell (HSPC) predominance and progression to MPN-blast phase. MKs are an important source of transforming growth factor β (TGFβ1). TGFβ1 has been implicated in the alteration of HSCs regeneration during stress conditions (8). TGFβ1 is the driver and primary mediator of changes in the tumor microenvironment and is a critical factor that limits the efficacy of checkpoint inhibitor therapy for patients with several types of solid tumors (9, 10). The events that directly drive TGFβ1 overproduction in MF remain unknown. Under pathologic conditions, abnormal MKs promote bone marrow (BM) fibrosis by releasing TGFβ1 and other pro-inflammatory cytokines as well as proteases due to a process termed emperipolesis which is a consequence of an aberrant distribution of MK-P-selectin which facilitates MKs engulfing other hematopoietic cells particularly neutrophils (11, 12). MKs can also influence HSC fate decisions and depletion of MK results in a reduction of HSC numbers (13, 14). Due to their elaboration of multiple cytokines and soluble mediators, MKs are also considered immunomodulatory cells which are capable of affecting the function of immune cell populations that change following the initiation of fibrosis (15). Accordingly, increased levels of pro-inflammatory cytokines secreted especially by MF MKs and monocytes play a pivotal role in MF disease progression by establishing an aberrant crosstalk between malignant and normal HSCs and the BM microenvironment. Overproduction of pro-inflammatory cytokines activate mesenchymal stromal cells (MSCs) that in turn participate in the amplification and differentiation of MF HSCs by further producing cytokines and matrix proteins. Therefore, MF is considered an inflammation-driven tumor where the inflammatory milieu contributes to genetic instability present in MF HSCs (16–18). Interactions between BM stroma and HSCs activate a cascade of pathways including NF-kB and MAP Kinases, which contribute to the maintenance of the ongoing inflammation. Breaking the vicious cycle of aberrant crosstalk between malignant MF HSCs and the BM microenvironment provides an avenue by which one can potentially limit the secretion of pro-inflammatory cytokines and therefore the expansion of malignant HSCs clones and MF disease progression.

In this review, we will discuss the contribution of MKs in promoting inflammation by releasing factors that sustain the proliferation of MF malignant clones and alter the hematopoietic microenvironment.

## Alterations in Myelofibrosis- Megakaryocyte Development

Aberrant megakaryopoiesis is a pathological hallmark of each of the MPNs (6). Several MPN mouse models have established the role of TGFβ in the development of BM fibrosis and have implicated MK hyperplasia and dysplasia, as key players in the creation of a characteristic MF inflammatory and metabolic signature (19–23). It is possible that alterations in MKs development occur during disease progression. In an inflammatory microenvironment, MK precursors can be directly produced not only by MK progenitor cells but also by MK-biased HSCs (24).

Inflammation induces stress megakaryopoiesis due to the upregulation of CD41, an early marker that defines more immature MKs expansion in the HSC compartment (24), suggesting that MF-MK hyperplasia is driven not only by aberrant JAK/STAT signaling but also by microenvironmental factors. It is increasingly understood that distinct cell populations can initiate or sustain the inflammation, which plays an important role in the origins of MPNs and their progression to blast phase (25–27). MPNs are characterized also by increased monocytes and macrophages production, which participate in the generation of the inflammatory milieu that contribute to the HSC niche in MF (28–30). By single cell RNA sequencing, Sun and coworkers showed that MKs are heterogeneous with populations that have distinct functions, one regulating HSCs, a second involved in pro-platelet formation, and a third with innate and adaptive immune functions (31). In MF, the expanded MKs secrete a large amount of pro-inflammatory and pro-fibrotic cytokines. More recently, Psaila and coworkers have reported that *JAK2V617F* mutant MF HSPCs are biased toward the MK lineage with the CD41 MK marker being expressed by both CD38+ and CD38- fractions of CD34+ cells as compared to the healthy donors (32). Moreover, MK precursors were characterized by an enrichment of inflammatory pathways in comparison to the healthy donors with an expansion of an aberrant MK population (32).

Anemia and thrombocytopenia due to ineffective erythropoiesis and megakaryopoiesis characterize patients with advanced MF (33). Megakaryopoiesis involves various transcription factors including GATA1, GATA2, FOG1 and NFE2. GATA1 and FOG1 promote MK and platelet differentiation (34, 35). The observed decrease of GATA1 content in MKs from MF patients or MKs with hypomorphic mutation in GATA1 (GATA1^low^) in an MF murine model, have implicated GATA1 as an important player in the progression of the MF (11) impairing the proper MK maturation (36). Mice that express low levels of GATA1 due to specific deletion of regulatory region or promoter sequences develop anemia and thrombocytopenia.

In human MF, hyperactive thrombopoietin (TPO) signaling leads to a deficiency of GATA1 expression contributing to impaired MK maturation (36). Similarly, mice treated with high concentrations of Tpo (Tpo^high^), are characterized by a decrease of GATA1 in MKs and simultaneously by an increase of TGFβ1 content in MKs contributing to the development of marrow fibrosis (11, 23, 37). Expression of GATA1 effectively can rescue MK maturation in MF indicating that GATA1 contributes to impaired megakaryopoiesis in MPNs (36). The mechanisms that link high levels of TPO signaling with reduced GATA1 content in MKs is unclear.

## Myelofibrosis Microenvironment and Clonal Hematopoiesis

HSCs are predominantly quiescent but they can be rapidly recruited into cell cycle by stress such as chemotherapy, infection, inflammation or bleeding. In MPNs, HSC mutations induce a selective growth advantage over normal HSCs due to MPN-HSCS being more responsive and sensitive to microenvironmental signals. The expanded abnormal myeloid clones, especially MKs and monocytes perturb the bone marrow (BM) microenvironment promoting a further self-reinforcing malignant niche that favors clonal amplification of MPN-HSCs at the expense of normal HSCs and eventual egress to the spleen and liver (38).

Cytokine overproduction in MPN mouse models has been found to be dependent on maintenance of enhanced NF-κB-dependent gene expression by BET bromodomain proteins (39) and on JAK/STAT activation in malignant and non-malignant clones (40). Although inhibition of JAK2 by the JAK1/2 inhibitor, ruxolitinib improves constitutional symptoms it does not affect the number of malignant HSCs or the degree of MF-BM fibrosis (41) suggesting that combination therapies targeting cytokine mediated signaling are required in order to enhance the MF-modifying potential of ruxolitinib.

Moreover, inflammatory mediators induce the accumulation of myeloid cells that are immunosuppressive. MKs from MF patients are characterized by elevated production of programmed cell death ligand (PDL-1) that induces T cell exhaustion by binding to the PD-1 receptor (42, 43). The PD-1/PD-L1 axis has been exploited to develop effective therapies for cancer patients with tumors that overexpress PD-L1 (44). Targeting PD-1 or PD-L1 alone has not proven sufficient to reverse the MF phenotype. Pembrolizumab, an FDA approved inhibitor of PD-1, has been used in MF patients without any clinical responses (45). TGF-β1, the isoform of TGFβ present at the highest levels in many human cancers contributes to anti-PD-1 resistance (46) by increasing PD-1 expression by CD8+ T cells (47). Increased levels of TGFβ1 may contribute to the T cells exhaustive state in MF patients. In advanced malignancies, TGFβ1 has been reported to increase the production of regulatory T cells (T_reg_) that inactivate helper T cells by inducing transcription factor FOXP3 (48) which is essential for programming T_reg_ cells development and function (49). Since TGFβ1 is also expressed on the membrane of T_reg_ cells, it inhibits cytotoxic T cell activity (50). This provides a biological rationale for combining inhibitors of PD-1 with TGFβ1 inhibitors for treating cancer patients, since they can target both PD-L1/PD-1 and the TGFβ pathway facilitating T cell penetration into the tumor microenvironment generating enhanced antitumor immunity and potentially reducing of the malignant cell burden (51). Accordingly, treatment with dual monoclonal antibody strategy with the capacity to inhibit TGFβ and PD-1/PD-L1 has resulted more effective than treatment with either PD-L1 antibody or TGFβ trap alone in increased T cells, NK cells, and decreased neutrophils and myeloid-derived suppressor in tumor-derived cells from syngeneic mouse model (52). Moreover, in preclinical studies the bi-functional blockade of PD-1/PD-L1 and TGFβ pathway has shown antitumor efficacy in patients with solid tumors leading to regression of colon and pancreatic adenocarcinoma in genetic mouse model (53–55).

## MEGAKARYOCYTES Are Important Components of the Hematopoietic Niche

MKs are a cellular component of the niche that contributes to the maintenance of the BM HSC pool size by promoting HSCs quiescence due to TGFβ and CXCL4 signaling (13, 14). TGFβ1 is expressed in MKs to a greater extent than other niche cell types and their selective deletion increases HSCs activation and proliferation through downregulation of pSMAD2/3 signaling (13). In fact, HSCs with nuclear localization of pSMAD2/3 lie in close proximity to MKs and the number of pSMAD2/3 LT-HSCs are reduced following MK depletion (13). These observations indicate that blunting of TGFβ signaling due to MK ablation might account for LT-HSCs activation. Moreover, HSCs co-culture with MKs promotes reconstitution of LT multi-lineage hematopoiesis while genetic deletion of MPL or treatment with blocking anti-CD41 antibody reduces HSC engraftment potential in irradiated mice (56).

During HSPCs regeneration, TGFβ1 signaling is transiently activated and experiments in mice indicate that blocking TGFβ1 signaling during stress conditions, accelerates HSC multi-lineage cellular reconstitution and delay the return of cycling HSCs to quiescence (13). TGFβ, due to its pleiotropic role has different effects, which are cell type specific. TGFβ1 induces the proliferation of human BM MSCs and the deposition of collagen mimicking the fibrotic futures of MSCs derived from MF (57). We have reported that patients with MF have dramatically increased levels of TGFβ1 from plasma and MK cultures in comparison to the healthy donors, which supports the predominance of MF-HSCs by altering the hematopoietic microenvironment and enhancing the quiescence of a reservoir of normal HSCs (58, 59). In comparison to TGFβ2 isoform, a positive regulator of normal hematopoiesis (60, 61), TGFβ1 and TGFβ3 inhibit normal hematopoiesis through the canonical SMAD-dependent signaling pathway (8, 13, 58). Treatment with a TGFβ1/3 specific protein trap (AVID200) restored normal hematopoiesis in MF patient samples that were responsive to TGFβ and reduced fibrosis in Gata1^low^ mice (58). More precisely, AVID200 treatment led to an increase in the number of HPCs with wild type *JAK2* by blocking TGFβ1-induced p57^Kip2^, GATA2, p21 expression and SMAD2 activation followed by a reduction of *JAK2V617F* mutated clones, suggesting that the specific TGFβ1/3 inhibition can restore normal hematopoiesis by allowing wild type HPSCs to exit quiescence (58). While this was pronounced in MF samples that were sensitive to TGFβ1, in resistant MF patient samples, AVID200 was not able to affect TGFβ downstream targets (58). HSPCs from *JAK2V167F^+^* patients have been reported to be characterized by a high degree of heterogeneity with different genetic sub-clones and sub-fractions of MK progenitors transcriptionally similar to healthy donors that can play a role in the MF evolution and therapy response (32). Our studies helped to confirm the existence of the heterogeneity in MF patient samples as regards to response to TGFβ and to elucidate the origin of resistance to AVID200 which might allow one to gain further insight into improved treatment approaches. We observed that in MF patient specimens resistant to TGFβ1, MK conditioned media contained higher levels of TGFβ1, accompanied by higher *JAK2V617F* allele burdens and by additional mutations that could have altered the TGFβ response (58). TGFβ has been previously reported to inhibit normal MKs production. In thrombocytopenic MF patients that were ineligible for ruxolitinib therapy, in a phase I clinical trial of AVID200 therapy led to substantial increases in platelet numbers and reduction of TGFβ1 serum levels indicating that TGFβ1 plays a pivotal role in MF associated thrombocytopenia which can be reversed with AVID200 therapy (59). However, 6 months of AVID200 in advanced MF patient population did not lead to reductions in BM fibrosis. These clinical data suggest that AVID200 might be best employed if administered for longer periods, at earlier stages of MF or in combination with another drug active in treating MF.

In addition,TGFβ activates MSCs which participate in the amplification and differentiation of hematopoietic clones as a consequence of altered HSC-niche cross talk. In human and murine MF, MSCs as fibrosis driving cells are characterized by a significant upregulation of JAK/STAT and TGFβ signaling pathways.

MSCs can be activated as well by inflammatory cytokines secreted by malignant HSC clones and once activated can become inflammatory imprinted and their activation causes an altered cross talk between HSCs and stroma within their BM niche (62). These data suggests that MSCs are important additional therapeutic targets in MF. We have reported that TGFβ1 from conditioned media derived from MF MK cultures induced proliferation of normal MSCs and collagen production and that AVID200 treatment was able to block MSCs proliferation and collagen production by blocking pSMAD2 and pJNKs activation pathways (58).

**Figure 1:**
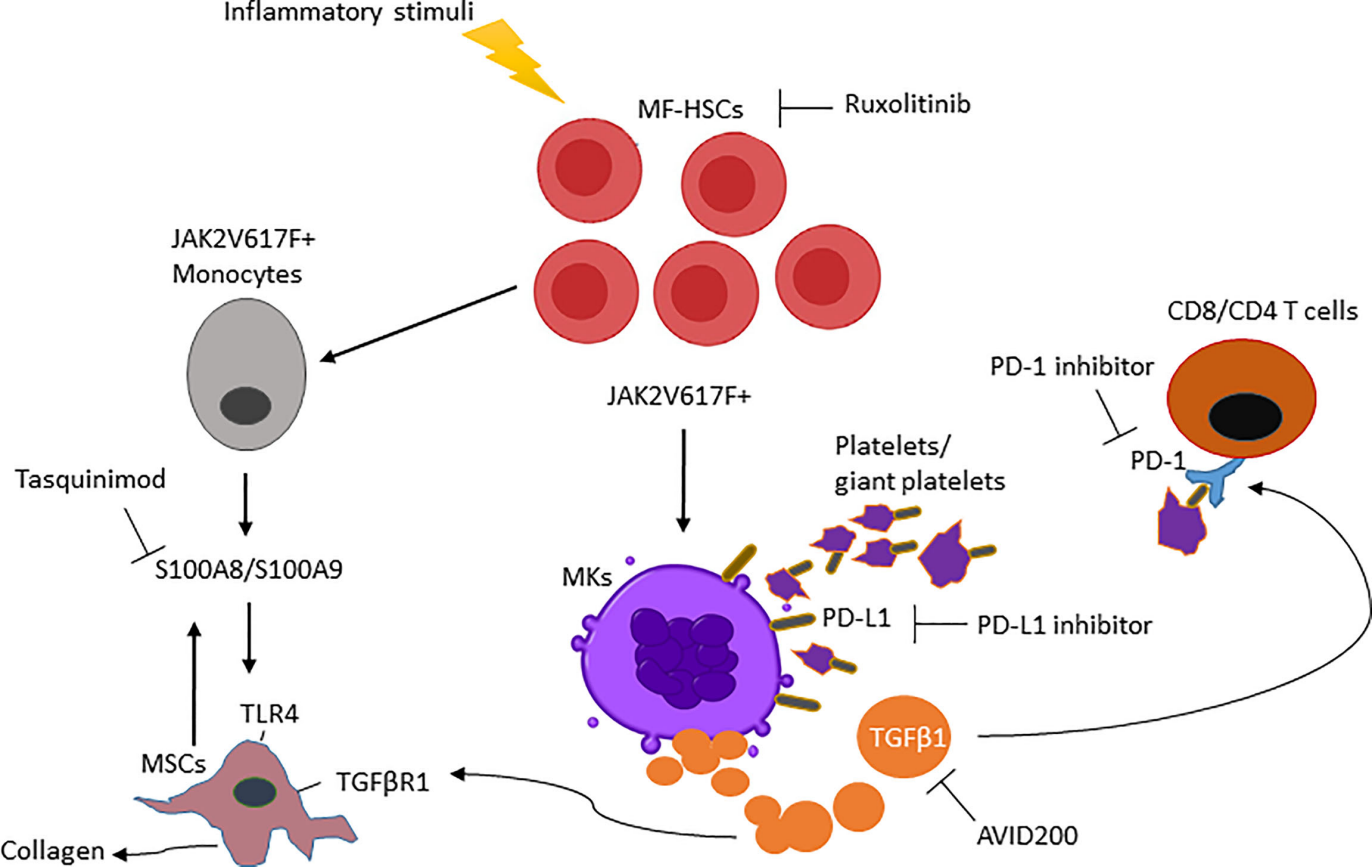
Main mediators in myelofibrosis and combination therapies are required to enhance the MF-modifying potential of ruxolitinib. (i) Clonal MF-HSCs events are favored by inflammatory stimuli. (ii) MF-HSCs will further differentiate into MKs and monocytes producing increased TGFβ1 and S100A8/S100A9 that modify the BM microenvironment and activate TGFβR1/TLR4 from MSCs. (iii) TGFβ1 ligand increases PD-1 expression on T cells and, PD-L1 expressed on the platelets interact with PD-1 on T cells causing its exhaustion. (iiv) As a result, the interruption of these MF-HSC mediators by a single pharmacologic inhibitor will be ineffective in reducing MF-HSCs unless used in combination therapy.

Ultimately, MSCs are the cell population responsible for initiating BM fibrosis in both murine and human MF. Based on transcriptomic pathway analyses during the course of the disease, MF-MSCs pre-fibrotic can be distinguished from overt fibrosis MF-MSCs. More precisely, two distinct subpopulations in the MSC compartment have been identified with adipogenic and osteogenic signatures respectively during the pre-fibrotic stage and at the time of acquisition of the fibrotic phenotype (63). The pre-fibrotic stage is characterized by a heterogeneous inflammatory signature. Conversely, overt fibrosis MSC subsets were characterized by higher expression of TGFβ1 as compared to pre-fibrotic MSCs (63), indicating that additional events likely precede TGFβ signaling in the biogenesis of MF. Moreover, single-cell RNA sequencing reveal that pre-fibrotic MSCs express the alarmin S100A8/S100A9 heterocomplex (63) which act through the inflammatory activating endogenous toll-like receptor 4 (TLR4) as a mediator of MF development prior to the time that TGFβ plays an active role (41). TLRs are critical mediators involved in innate immune signaling and are overexpressed in HSCs isolated from MF patients where chronic inflammation, pro-inflammatory cytokines and immune defects are linked to the development of MF. Fibronectin can stimulate TLR4 production contributing to cell proliferation and dysregulated megakaryopoiesis. Alarmins, secreted by MSCs and HSCs, are also associated with activation of NOD-like receptor protein 3 inflammasome, leading to ineffective hematopoiesis, DNA damage and consequent production of IL-1β and IL-18 by HSC progenitors (64–66). These data suggest that mutated HSCs can initiate a vicious cycle of inflammation within the stem niche, leading to decreased support of normal hematopoiesis and sustaining the progression of HSCs malignancy. The expression of alarmins in MF has been associated with progression toward the fibrotic phase and their activity can be blocked by a small molecule inhibitor tasquinimod that impedes alarmin interaction with TLR4 leading to a significant reduction of the MF phenotype (63). These data raise the possibility that tasquinimod or a similar acting drug can target the tumor microenvironment and in combination with immunotherapeutic agents can increase tumor immune responses. Overall, our increased understanding of MK development may lead to more precise therapeutic strategies that can be used to target MF-HSCs and stroma at different stages of the disease (**Figure 1**).

## Conclusion

The MPNs are clonal hematopoietic disorders with profound degrees of systemic inflammation. The release of inflammatory mediators by clonal hematopoietic cell populations remodels the HSC compartment promoting the emergence of disease and progression. It is crucial that future studies focus on better understanding how inflammation and innate immune pathways regulate the interactions of HSCs with the HSC niche both during normal and malignancy-associated hematopoiesis.

Conventional treatments for MF are focused on controlling symptoms and have limited MPN-disease modifying potential. Due to the critical role that MKs play in MF, clinical trials that can more optimally target the dysregulated MKs are needed. Numerous trials are being developed to assess whether targeting the inflammatory pathways can modify MF disease progression. MF with advanced marrow fibrosis is associated with excessive production of fibrogenic, pro-inflammatory cytokines and cell contact mediated inflammatory activators, and therefore interruption of vicious inflammatory cycle with a single agent therapy seems unlikely. Therapeutic approaches targeting MKs might be more successful if used early on in the course of the disease preventing MK/MF HSC interactions as well as differentiation of pro-fibrotic stromal cell populations and their interaction with hematopoietic clones.

## Author Contributions

LV and RH conceptualized and designed the outline for the manuscript, wrote, edited and approved the manuscript.

## Funding

This work was supported by the National Cancer Institute Program Project grant P01CA108671.

## Conflict of Interest

The authors declare that the research was conducted with the financial support of Forbius who provided AVID200 and research support for the preclinical studies as well as partial support for the clinical trial of AVID200, which was used to assist with regulatory matters. Forbius did not influence the outcomes of these studies.

## Publisher’s Note

All claims expressed in this article are solely those of the authors and do not necessarily represent those of their affiliated organizations, or those of the publisher, the editors and the reviewers. Any product that may be evaluated in this article, or claim that may be made by its manufacturer, is not guaranteed or endorsed by the publisher.
